# Process Analytical Technology Tools for Monitoring Pharmaceutical Unit Operations: A Control Strategy for Continuous Process Verification

**DOI:** 10.3390/pharmaceutics13060919

**Published:** 2021-06-21

**Authors:** Eun Ji Kim, Ji Hyeon Kim, Min-Soo Kim, Seong Hoon Jeong, Du Hyung Choi

**Affiliations:** 1 Department of Pharmaceutical Engineering, Inje University, Gimhae-si, Gyeongnam 621-749, Korea; k89321499@gmail.com (E.J.K.); gihyeon906@gmail.com (J.H.K.); 2 College of Pharmacy, Pusan National University, Busandaehak-ro 63 heon-gil, Geumjeong-gu, Busan 46241, Korea; minsookim@pusan.ac.kr; 3 College of Pharmacy, Dongguk University-Seoul, Dongguk-ro-32, Ilsan-Donggu, Goyang 10326, Korea; shjeong@dongguk.edu

**Keywords:** process analytical technology, continuous process verification, quality by design, control strategy, quality attributes, critical process parameters

## Abstract

Various frameworks and methods, such as quality by design (QbD), real time release test (RTRT), and continuous process verification (CPV), have been introduced to improve drug product quality in the pharmaceutical industry. The methods recognize that an appropriate combination of process controls and predefined material attributes and intermediate quality attributes (IQAs) during processing may provide greater assurance of product quality than end-product testing. The efficient analysis method to monitor the relationship between process and quality should be used. Process analytical technology (PAT) was introduced to analyze IQAs during the process of establishing regulatory specifications and facilitating continuous manufacturing improvement. Although PAT was introduced in the pharmaceutical industry in the early 21st century, new PAT tools have been introduced during the last 20 years. In this review, we present the recent pharmaceutical PAT tools and their application in pharmaceutical unit operations. Based on unit operations, the significant IQAs monitored by PAT are presented to establish a control strategy for CPV and real time release testing (RTRT). In addition, the equipment type used in unit operation, PAT tools, multivariate statistical tools, and mathematical preprocessing are introduced, along with relevant literature. This review suggests that various PAT tools are rapidly advancing, and various IQAs are efficiently and precisely monitored in the pharmaceutical industry. Therefore, PAT could be a fundamental tool for the present QbD and CPV to improve drug product quality.

## 1. Introduction

Quality control in the pharmaceutical industry has traditionally depended on statistical process control (SPC) [1,2,3,4], which is used to understand the process and desired specification limits and to ensure a stable process by eliminating the allocable sources of variation. Statistical methods, including control charts and run charts, are used to inspect the quality of the post-manufacturing finished product and determine the performance suitability of unit operations in the pharmaceutical manufacturing process [1]. Moreover, most offline analyses and monitoring are conducted to evaluate the quality of the intermediate and finished products during the production batch process. For example, it is common to use control charts for monitoring general production processes, thereby ensuring that various aspects of the production process are controlled [5,6]. This traditional process verification is designed to perform process verification on finished batches under predesigned process conditions. Therefore, a disadvantage of this method is that the quality characteristics of intermediate products cannot be confirmed during the manufacturing process. Hence, identifying and solving problems that arise during the process requires a lot of time and results in relatively more high-quality defects. Moreover, there is no assurance that the entire lot conforms to the required specifications, and the method cannot be applied generally as a solution to all quality defects.

The International Council for Harmonisation (ICH) launched continuous process verification (CPV) to overcome SPC limitations, ensure process control, and improve the understanding of processes and product quality. Furthermore, ICH described CPV as an alternative approach to process validation, in which manufacturing process performance is continuously monitored and evaluated. In addition, CPV provides more information about variability and control, providing higher statistical confidence, improving the assessment of pharmaceutical manufacturing processes and higher assurance of continuous control status.

Another strategy introduced by the pharmaceutical industry to improve the understanding of the process and quality control is quality by design (QbD). QbD is defined in ICH Q8 guidelines as “a systematic approach to development that begins with predefined objectives and emphasizes product and process understanding and process control, based on sound science and quality risk management.” The development of a QbD-based pharmaceutical process involves a scientific risk-based systematic method to correlate critical process parameters (CPPs), input-materials attributes, and critical-quality attributes (CQAs) [3]. In general, QbD tools, including design of experiments (DoE), empirical modeling, and response surface analysis can develop a design space and reveal process variability during the pharmaceutical manufacturing process [7,8,9]. Unlike the existing quality by testing (QbT) system, in which the quality test of the finished product is mainly used, the QbD approach enables drug-quality management to enhance the quality of drugs based on science- and risk-based technology.

The US Food and Drug Administration (FDA)’s Center for Drug Evaluation and Research (CDER) discussed the need for FDA guidance to facilitate PAT implementation, and the FDA published the PAT guidance for innovative pharmaceutical manufacturing and quality in September 2004 [10]. It is recognized as an important paradigm shift in inspecting and approving processes for the continuous process verification of pharmaceutical production processes. This initiative is also implemented by the EMA, and the Ministry of Health, Labor, and Welfare (MHLW) in Japan adopted it immediately [11]. Interfacing manufacturing processes with analytical techniques is essential in PAT, as it facilitates process development according to QbD principles and enables real-time release testing (RTRT) [12]. PAT is applied to each unit operation in the manufacturing process; CPPs, which have a significant influence on CQAs, are controlled to present a high-quality product in the market [13,14,15].

PAT in CPV ensures product quality throughout the manufacturing process and enables the automation of transportation between product processes [16,17]. Furthermore, PAT is used as a control strategy for monitoring processes in real time, improving the understanding of the process, and RTRT [11,18,19]. The vast amount of information obtained by PAT enables rapid problem resolution, optimization, and defect detection. In addition, in the event of unexpected process changes, PAT can be applied to identify the root causes of undesired drug product-quality issues. Therefore, appropriate PAT enables the timely adjustment of process parameters, ensures good and stable product quality, and shortens the overall manufacturing time. These frameworks provide advantages that enable process control quickly and easily and are a trend that has been gradually adopted and introduced because it contributes significantly to establishing the control technology [18,19,20,21]. Furthermore, several studies have applied the QbD approach and PAT in pharmaceutical manufacturing processes [12,14,16,17,18].

This review focuses on applying PAT to QbD, RTRT, and CPV to improve drug quality in the pharmaceutical industry. It presents a significant relationship between the process and IQAs with the relevant literature, which could be monitored with PAT framework for QbD, RTRT, and CPV. The recent PAT tools are presented with the relevant literature in pharmaceutical unit operations, including blending, granulation, tableting, and coating. Based on unit operations, IQAs measured by PAT, equipment type, PAT tools, multivariate statistical tools, and mathematical preprocessing are listed along with relevant literature.

## 2. Control Strategy for PAT Application

Appropriate control strategies should be applied during the manufacturing process to control variables affecting product quality. A control strategy comes from the understanding of products and processes and risk management. There are various approaches, such as in-process testing, RTRT, and finished product testing [11,14,15]. Traditional control strategies mostly rely on off-line analysis of finished-product testing. In addition, process verification has been performed on batches produced under predesigned process conditions. However, because it is difficult to predict the effect of process parameters during processing on finished-product quality, there is a limit to effectively controlling the process. It cannot be determined that all produced lots comply with the requirements. In addition, it is not easy to establish the feasibility of controlling the process variables of each unit process. Therefore, real-time process control is impossible and inefficient in terms of time and cost. The QbD approach has been introduced to overcome this and to improve understanding of product performance, identify critical process parameters (CPPs) during quality risk assessment of the product manufacturing process, and establish appropriate control strategies for each variable [13,22]. The QbD approach is applied for the accurate and reliable prediction of product-quality characteristics within the design space established, using each variable, manufacturing environment, and other conditions [12]. As this improves the understanding of products and processes, control strategies are applied to produce products of consistent quality that meet the desired quality attributes [23,24]. Introducing process control strategies to minimize the variability of the finished-product quality can justify an approach to quality assurance with an improved level of quality compared to finished-product testing using existing compendial standards.

### 2.1. The Effect of the Manufacturing Process on Intermediates during Processing

As described above, CPV was introduced in the pharmaceutical industry to produce high-quality drugs through quality control and quality assurance throughout the drug lifecycle. Therefore, in CPV, the quality control and process monitoring of intermediate products are recommended by using QbD to identify the quality of intermediate products that may affect the quality of finished products and by adjusting process parameters during the manufacturing process using the PAT framework. [12,13,22]. Table 1 presents the process parameters and quality of intermediate products that need to be adjusted in the manufacturing process, including blending, granulation, drying, coating, and tableting of solid dosage form based on the risk assessment using the QbD approach. Since the proposed process parameters and quality of the intermediate can greatly influence the quality attribute of the finished product, they should be adjusted by conducting appropriate process monitoring through a PAT framework during the manufacturing process [23,24].

### 2.2. Workflow of PAT Framework for the Pharmaceutical Manufacturing Process

Before applying PAT to the process, first, it is necessary to understand the process and materials and consider the characteristics of the PAT tool. The most appropriate PAT tool is selected and applied to the PAT process. In this process, factors such as the location of the PAT tool and the measurement method should be considered. The measurement methods of PAT during the process are classified into at-line, on-line, and in-line. The at-line method is a measurement method that collects, separates, and analyzes a sample from a place very close to the process. On-line is a measurement method in which a sample is measured in the manufacturing process, suitability is determined, and the sample is returned to the process or discarded. In-line is a real time monitoring method using software without collecting a sample from the process flow [121]. After that, process monitoring is performed, and the collected data is analyzed and evaluated with statistical methods. The statistical methods could be divided into preprocessing technologies, chemometric modeling, and data evaluation. Standard normal variate (SNV), multiplicative scatter correction (MSC), and derivatives to reduce data interference and correct data are used in the preprocessing step [60,122,123,124,125]. The chemometric modeling includes partial least square (PLS), principal component analysis (PCA), multiple linear regression (MLR), etc., to confirm the correlation between CQAs, critical material attributes (CMAs), and CPPs [16,20]. Data evaluation is to measure and enhance data predictability using the root mean square error of the calibration (RMSEC), root mean square error of the prediction (RMSEP), etc. [126,127,128]. Various literature presented use PAT in this way. It is suggested that process control and quality control can be performed by measuring the quality of intermediate and finished products in RTRT using PAT during the process.

### 2.3. The Role of PAT Framework on QbD, CPV, and RTRT

**Figure 1:**
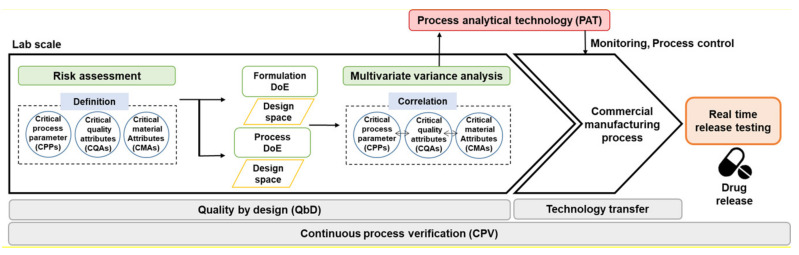
Framework of process analytical technology (PAT) application in quality by design (QbD) approach.

As shown Figure 1, PAT is important for CPV and QbD approaches to the production of high-quality drug products. In lab-scale drug development, CMAs, CPPs, and CQAs in the formulation and process are identified through QTPP and risk assessment, an optimal design space is derived through DoE, and correlations with CQAs, CPPs, and IQAs are identified through multivariate analysis (MVA) based on QbD [35]. Based on the correlation between CQAs, CPPs, and IQAs, process and quality control are possible by checking process variability in real-time through PAT during the commercial-/pilot-scale manufacturing process. Therefore, the introduction of PAT in the QbD approach in the pharmaceutical manufacturing process is used as a control strategy for RTRT by improving process understanding through monitoring the process in real-time and enabling rapid identification and response [11,129,130,131,132,133]. In other words, performing RTRT using PAT application in real time to manage the correlation identified based on the QbD approach enables CPV through the production of high-quality drug products with guaranteed product qualification throughout the manufacturing process.

## 3. PAT Tools for the Pharmaceutical Manufacturing Process

### 3.1. Near-Infrared Spectroscopy (NIRS)

After discovery by Herschel in the 1800s, NIRS has been applied as a useful spectral analysis technology in many studies. NIRS is a qualitative and quantitative analysis based on the transmittance and reflectance generated by molecular vibrational motion using light in the near-infrared range of 780–2800 nm. It is most often used in the pharmaceutical industry as a real-time process-monitoring tool for product quality control and quality assurance during processing [11,19,20,21,22,23,24]. The NIRS is connected to a fiber optic probe, and the QA of the product in the process is non-destructively measured by the transmission and reflection of the NIRS by the sample, and quality control through real-time monitoring is possible [19]. The inside of the probe is composed of an optical fiber, a lens, mirror, and signal channel, and the outside of the probe is made of a corrosion-resistant material. It is connected through a sapphire window, so it can be effectively used even in poor process conditions. When the probe is connected to the NIR spectrometer, the light emitted from the light source is focused by the focusing lens. The light reflected by the mirror located at the tip of the probe is transmitted to the NIR spectrum by reflecting a sample. The transmitted signal forms a spectrum through computer software connected to the NIR spectrometer. However, a disadvantage of NIRS is that it is more difficult to interpret a signal than by using conventional analysis methods, such as chromatography, ultraviolet/visible (UV-VIS) light, and others, because the absorption bands overlap due to spectral complexity. In addition, because this is a relative approach, it is necessary to form and verify an accurate correction model using a reference method to utilize it effectively [20]. Nevertheless, NIRS can non-destructively measure the IQAs of a product in a short time during the process, and it can be used as a tool for RTRT in the pharmaceutical industry by enabling the process control and quality assurance of finished products through real-time monitoring [7,21,72,134,135,136,137,138,139,140,141,142,143,144,145,146,147,148,149,150,151,152,153,154,155,156]. Some literature, which shows that PAT is highly applicable to CPV through RTRT, conducted to monitor and evaluate product quality by applying NIRS as a PAT tool, are presented below in various pharmaceutical industries.

### 3.2. Raman Spectroscopy

Similar to NIRS, Raman spectroscopy is a noncontact analysis technology that uses optical fibers [157]. Raman spectroscopy is a type of vibration spectroscopy. Various Raman laser sources offer a range of wavelengths (generally 785 nm), from the UV-VIS to near-infrared regions; the most common are visible light lasers [158]. In general, vibrations occur in chemical bonds that are not rigid, and materials can be characterized based on their molecular-vibration frequencies. Raman spectroscopy is widely used in pharmaceutical manufacturing because it enables the rapid characterization of the chemical composition and structure of a solid, liquid, gas, gel, or powder sample by providing the detailed characteristics of their vibrational transitions [1]. Raman spectroscopy is used to determine the molecule in the sample, and their intensity enables the calculation of the drug content of a particular sample. One of the main reasons for using PAT is to build and qualitatively analyze the specificity library of the raw material spectrum, including impurities in the sample [158]. Raman spectroscopy is ideally suited for PAT systems because it has the flexibility to operate on-line or in-line. Moreover, it provides both quantitative and qualitative data, enabling accurate and consistent monitoring and control during real-time processes. Depending on the compound, the Raman spectrum for a specific molecule differs for each movement of the scattered photon energy, and because it has a unique fingerprint, it enables the monitoring of qualitative information [8,159]. Furthermore, it can be used for analyzing liquid products without moisture interference, similar to Fourier-transform infrared spectroscopy (FTIR) or NIRS, and has a high measurement speed.

Similar to other spectroscopy methods, Raman spectroscopy is commonly used as a real-time monitoring tool for CPV in various pharmaceutical unit processes, including blending, granulation, coating, and tableting. It can analyze the IQAs and CQAs of drug content [160,161,162] during the blending process, moisture content [8,9] during the drying and granulation process, and coating thickness and content [157,163,164] during the coating process, as well as enable polymorph identification in API preparation [165,166,167], granule-formulation analysis [8], blending uniformity [27,168], particle-size analysis [16,159,169], and others.

### 3.3. Hyperspectral Imaging (HSI)

HSI is well known for chemical or spectral imaging. It is a nondestructive PAT tool that can extract both spatial and spectral information from an object by integrating existing imaging and spectroscopy techniques. HSI can be applied to various wavelength ranges, including visual, near-infrared, and short-wave infrared (1000–2500 nm). Each pixel of the image acquired by the HSI tool, which contains a spectrum of a specific location, comprises hundreds of consecutive wavelength bands in each space. This generates a large amount of information because the spectrum is constantly acquired from a wide range. Similar to other spectroscopy methods, the preprocessing of the acquired data cube must be performed on qualitative and quantitative images by extracting information in an easy-to-understand image format [170]. HSI provides dependable chemical and spatial imaging data on the content and distribution of API and excipients during the processes of blending, granulation, and tableting [170]. The acquired images are combined and processed in three dimensions (3D) to form the data cube. The x and y dimensions of the formed cube are shown as the two space dimensions, whereas λ is the spectral dimension. There are four basic techniques (spatial scanning, spectral scanning, nonscan, and spatiotemporal scanning) for acquiring 3D (x, y, λ) data in a hyperspectral cube, and the choice of technology depends on the specific application. HIS as an on-line PAT tool has been used to monitor blending uniformity and analyze tablet variability [171]. Moreover, it can analyze the sample to be measured faster than spectroscopy, and it is also used for package monitoring to ensure products are correctly placed in the package, identify defective tablets, or detect empty slots in a package [172]. Kandpal et al. studied an in-line HIS system for monitoring drug content in microtablets’ surface. The collected multivariate data were evaluated by applying the PLSR and PCR chemometric model. The authors showed a high predictive ability and proposed a quick in-line determination of product quality using HIS.

### 3.4. Terahertz Pulse Imaging (TPI)

TPI, a widely used PAT tool for real-time imaging, is applied for monitoring tablet surface and coating analysis [173]. The terahertz absorption spectrum is related to the 3D arrangement and covers the spectral range of 0.1–4.0 THz, which corresponds to the range between the infrared and microwave frequencies. For this reason, the terahertz region is known as far-infrared radiation. Compared with infrared radiation, it has the advantage of causing little scattering because of its longer wavelength, and lower radiation energy interacting with drugs is less likely to damage the sample [173]. Furthermore, TPI is widely used as a noninvasive method because it uses nonionizing radiation and is safe to use. As mentioned previously, TPI is mainly used in the pharmaceutical coating process. In particular, it is used for predicting the degree of coating thickness in sustained-release tablets, in which the coating thickness is directly related to drug release [164,174,175,176]. If drug release is via the coating instead of through the dissolution of the tablet, then it can be predicted by analyzing the coating formulation using TPI.

### 3.5. Mass Spectrometry (MS)

MS is an extremely useful PAT tool for the qualitative analysis of drug, compound, and related substances. Because it has a high resolution and mass accuracy, it is also used in the qualitative analysis of small molecules and is often selected and used in biological processes, such as in analyzing heterogeneous biomolecules [177,178,179]. In addition, it provides quick analysis when high throughput sample preparation and automated data processing are possible [8]. The mass spectrum is commonly employed to obtain the identity of two compounds or to establish the structure of a new compound and provides the accurate molecular weight or molecular formula to indicate the existence of a specific structural unit in a molecule. The main advantage of MS is its ability to measure several types of compounds with excellent discrimination over a very short analysis time. Moreover, it is used to quantitatively analyze known substances or identify unknown compounds in a sample and to reveal the structure and chemical properties of other molecules. To perform MS, a vacuum must be maintained, and the sample needs to be vaporized and ionized. Thus, the disadvantage of MS is that a sample cannot be analyzed if it cannot be decomposed and evaporated. The typical applications of MS include the real-time control of the drying process, particularly the monitoring of the trace amounts of organic solvents used in the production of intermediate and finished products.

### 3.6. Acoustic Resonance Spectrometry (ARS)

ARS as a PAT tool detects and analyzes the sound generated during the pharmaceutical process. The sound detected by ARS is much higher than the frequencies detectable by the human ear [180,181]. It is usually applied to processes that cause acoustic emission and is applied to the chemical reaction checking or blending, pulverization, and fluidized-bed granulation of the drugs. For example, during the granulation process, particles emit various sounds when they collide with each other and cause friction in the equipment. As with most PAT tools, ARS is noninvasive, does not require sample preparation, and has the advantage of being an inexpensive and convenient application method. Using acoustic emission, quantitative information, such as particle characteristics and moisture content, can be obtained. Changes in the physical properties of the powder, such as compression characteristics and distribution characteristics, can be monitored. Tsujimoto et al. used ARS to monitor and characterize particle motion due to friction occurring during the fluidized-bed granulation process; they also monitored the behavior of particles via the correlation between ARS and particle motion. ARS was installed at the bottom of the fluidized-bed granulator, and the collected sound was amplified and the sensitivity optimized to analyze the frequency. The impact of particles hitting the chamber wall increased with the increasing rotational speed of the fluidized-bed granulator, resulting in the subsequent increase of the AE amplitude. In addition, the instability due to the increased amount of spray solution, i.e., increased moisture content, could be detected during the fluidized-bed granulation process by ARS [180].

### 3.7. Spatial Filter Velocimetry (SFV)

Similar to FBRM, SFV is a technique that measures the chord length of a moving particle in real time. Therefore, it is used as a PAT tool for the real-time monitoring of particle size, size distribution, and shape in various solid dosage manufacturing processes, including fluidized-bed granulation/coating, grinding, and spray-drying [147,153,182,183,184]. However, unlike FBRM, which uses backscattered laser light, SFV applies a shadow to calculate the particle code length. When the particles pass through a parallel laser beam, a shadow is created in the linear fiber-optic array, and a secondary pulse signal is generated by a single fiber. Hence, it is possible to measure the size and velocity of individual particles simultaneously and calculate the chord length of the particle by using the time of the pulse signal and velocity of the moving particle [77]. Therefore, monitoring using SFV allows quality control to be performed by evaluating the properties of intermediate and finished products in a non-invasive method without special sampling procedures. Due to these characteristics, SFV can be used as a monitoring tool in CPV through RTRT.

### 3.8. Focused Beam Reflectance Measurement (FBRM)

FBRM is a technique that provides information on the code length distribution of a population of dispersed particles based on the backscattering of laser light. In the pharmaceutical industry, it is suitable for studying particle properties in suspension [185,186,187,188], emulsion [189,190,191,192], and crystallization [193,194]. Therefore, FBRM is used as a tool to evaluate IQAs including particle size, size distribution, shape, and particle-growth behavior in granulation and crystallization processes, which can have a great influence on the quality of the finished product due to particle properties, and to perform real time monitoring [195]. In the case of FBRM, a laser beam connected to the probe via a fiber is inserted into the process equipment through a sapphire window at the end of the probe. At this time, some of the light scattering generated by the laser beam crossing the particles by high-speed rotational motion is transmitted to the detector to generate the code length. Thousands of code lengths are measured simultaneously, and based on this, a code-length distribution can be generated to measure particle properties such as particle size and size distribution in real-time [92,93,95].

### 3.9. X-ray Fluorescence (XRF)

XRF is an atomic analysis technique used to determine the component of a variety of sample types, including solids, liquids, slurries, and powders, similar to inductively coupled plasma light atomic emission spectroscopy (ICP-AES) and atomic absorption spectroscopy (AAS). AAS and ICP-AES are widely used in the pharmaceutical industry for atomic high-sensitivity analysis because they can measure >70 different elements. However, only a specific analyte can be measured by the cathode lamp, and sample preparation takes longer because of the acid-decomposition procedure. Moreover, there are obvious disadvantages because of the large space requirement and high maintenance cost. As an alternative, XRF was developed [196]. XRF is a chemical analysis method based on the transfer of internal electrons and the interaction of X-ray radiation and atoms. High-energy X-rays attack electrons in high-energy atoms, leading to their release [16]. Hence, a vacancy is created in the inner shell, and electrons in the outer orbit are moved to cover the vacancy, thereby generating fluorescent X-rays because of the energy difference between the two orbits. Because each element has an electron of its own energy level, elemental analysis is possible as a result of the unique energy difference resulting from the characteristic X-ray irradiation [197]. Therefore, XRF has the advantage of high selectivity, a small number of collected spectra, and a lack of overlap [198]. The obtained spectrum of XRF indicates the properties of each element, and the intensity of the spectrum indicates the content of the element present in the sample [196]. XRF is unaffected by the matrix effect because it reduces the absorption and scattering of the X-ray beam between the sample and the matrix. Moreover, the sample preparation time is short, and the method is relatively simple because of its high sensitivity [199]. XRF is highly applicable to CPV in the pharmaceutical industry as it allows simpler data analysis because of its lesser influence on the non-overlapping spectrum and matrix effect, as well as its ability to nondestructively quantify multiple elements at the same time. This is explained through research cases in which the quality evaluation of intermediate and finished products was performed through the real-time monitoring of various manufacturing processes in the pharmaceutical industry.

### 3.10. Other PAT Tools

OCT (optical coherence tomography) is a high-resolution imaging method that can non-destructively measure the depth of translucent or cloudy materials. In the pharmaceutical industry, it is used to measure coating thickness and uniformity during the coating process by applying the same principles as TPI. OCT can compensate for the shortcomings of TPI’s low resolution and long measurement time and enables high resolution due to its relatively short wavelength [200,201]. However, imaging the thickness of the coating can be difficult due to strong scattering that limits the depth of penetration into the coating matrix and does not produce a clear refractive index difference. Nevertheless, it can measure not only the coating thickness but also the coating homogeneity within the tablet and has the advantage of being less affected by probe contamination and measurement location during the process compared to NIRS or Raman spectroscopy. Therefore, in the pharmaceutical industry, OCT is widely used as a real-time monitoring tool for the quality control of intermediate and finished products, and several studies have applied this tool to RTRT to prove the possibility of CPV [200]. Eyecon is a direct imaging analyzer of particle size. It does not require sampling and automatically captures data regarding the particle size and shape of the powder or variations to analyze the process. Another PAT tool is microwave resonance technology (MRT). MRT can measure moisture during the granulation process by noting the interaction between water molecules and the changing electromagnetic field. Unlike spectroscopy, such as NIRS and Raman spectroscopy, MRT does not require any mathematical preprocessing of the collected data.

## 4. Application of PAT Framework for Control Strategy of the Unit Operations

### 4.1. Blending Process

Blending is an essential process for preparing all formulations, including emulsions, suspensions, and injections, as well as solid dosage forms, such as granules, tablets, and capsules. Its main purpose is to obtain a homogeneous mixture of the API and excipients [202].

#### 4.1.1. Monitored IQAs in Blending Process

Unnecessary blending over long periods of time can change the particle size and size distribution because of wear. Such changes in the properties of intermediate products can greatly affect the quality of the finished products, such as assay, content uniformity, and dissolution. Therefore, during the process, it is necessary to inspect the IQAs, including blending uniformity, particle size, and particle-size distribution. Additionally, in this process, CPPs, such as blender speed and blender time, should be considered through real-time monitoring.

#### 4.1.2. Application of PAT Framework in the Blending Process for CPV

Existing offline analysis to evaluate blending uniformity is performed through sampling by stopping the process. However, most PAT tools are destructive, time-consuming, and often interfere with sampling. Thus, in the blending process, PAT tools can be used to nondestructively measure IQAs and process performance in real-time and to control CPPs through real-time monitoring for ensuring CQAs [19,121]. Next, the research that performed quality control by controlling IQAs and CPPs through monitoring using a PAT tool in blending process is presented in Table 2. In this literature, the possibility of utilizing PAT in CPV through RTRT is presented. El-Hagrasy et al. installed NIRS probes at six different locations in a V-blender to measure the powder blending uniformity and monitored the blending process using an InSb imaging camera. This was performed by adjusting the blending time of the blending process to measure the blending uniformity, and offline NIRS and UV-VIS measurement data were used as a reference method. The measured data were subjected to data preprocessing using second-order differentiation, SNV, and MSC to remove linear baseline shifts to obtain accurate data [203]. Also, Shi et al. used NIRS at various locations in Blender to monitor drug content [121]. This literature shows that monitoring at multiple sampling locations using a probe is essential for an accurate estimation of the PAT tool’s blending uniformity. In addition, since blending for an unnecessarily long time in the blending process affects the particle size, drug content, and particle-size distribution by reducing the blending uniformity, the necessity of monitoring during the process was confirmed. Martínez et al. developed a method to quantify the drug content, collected by monitoring with a NIR probe during the blending process using a continuous blender. The blending uniformity of the powder was measured by controlling the blending speed as CPPs [204]. Lee et al. predicted the particle-size distribution of excipients through real-time monitoring using in-line NIRS during the blending process. Performing PLSR modeling confirmed that the particle size correlated with the spectrum obtained through NIR monitoring. It can be used to predict particle size in real-time during the blending process using NIRS to quickly detect a problematic particle-size-distribution fluctuation to reduce incomplete blending [120]. Nagy et al. demonstrated that, during the continuous blending process, Raman spectroscopy is effective in monitoring and controlling drug content in real time. Drug content and blending uniformity measured through real time monitoring were quantified using the PLS model. Modeling was performed over a spectral range of 291–1490 cm^−1^, and all spectra were preprocessed, and the calibration dataset was validated with an appropriate cross-validation method. This study shows that, with the proposed PAT strategy, IQAs can be measured and predicted through in-line Raman spectroscopy monitoring, and technical malfunctions during the processing can be detected. It also argues that the proper PAT application is essential for CPV in the pharmaceutical industry [168]. In addition, De Beer et al. used Raman spectroscopy and NIRS simultaneously in a high-shear mixer in an in-line method to determine the endpoint of the mixing process through real-time monitoring. This study shows that, when two PAT tools are used simultaneously, they can be mutually verified, and thus, higher accuracy can be provided [59].

### 4.2. Granulation Process

Granulation involves the enlargement of powder particles via agglomeration technology, which secures the flowability of the powder, prevents segregation, and improves the compressibility of the powder during tableting [214]. It can be classified into dry granulation and wet granulation. Wet granulation is performed by spraying a liquid binder onto the particles while they are blended in a high-shear mixer, twin-screw mixer, or fluidized-bed granulator [215]. Unlike wet granulation, dry granulation does not use water or organic solvents; hence, it is particularly useful for drugs that are sensitive to moisture or heat [216]. Because the granulation process involves a complex mechanism, it is difficult to control and manage quality consistently, owing to the existence of many variables during the process. Therefore, it is important to use PAT to control CPPs in real-time during the granulation process. PAT monitoring is used for accurate and efficient quality control by quickly identifying and solving variabilities in granule/ribbon quality and CPPs, which is also highly applicable to CPV [141]. Studies have reported that quality control can be continuously performed by checking IQAs in real-time and adjusting process parameters [151]. However, an accurate measurement may not be possible because of contamination of the probe window due to the addition of a binder during the process, which is a disadvantage. To solve this problem, several studies have studied different probe positions during processing [8,142,143,144,145].

#### 4.2.1. Monitored IQAs in Granulation Process

In the wet granulation process, the particle size and size distribution of the granules have a decisive influence on the finished product’s CQAs, such as content uniformity, tensile strength, and friability. Thus, it is necessary to control granule aggregation and particle-growth behavior [214,215]. Moreover, moisture content affects CQAs, including the fluidity, stability, and compressibility of the finished product. Therefore, it is necessary to control moisture content during the process appropriately. Therefore, IQAs, such as the particle size and moisture content of the granules, must be monitored during wet granulation. During the high-shear granulation process, monitoring for quality control of intermediate product IQAS, such as granule growth, particle size, and moisture content is necessary by adjusting process parameters, such as binder content, impeller speed, and massing time.

Additionally, CQAs, such as ribbon density, particle size, particle-size distribution, and tensile strength, should be appropriately managed throughout the dry-granulation process. During a twin-screw process, the quality control of intermediate-product IQAS such as varying granule size, fluidity, and particle-size distribution in the finished product is important through controlling process parameters, such as the liquid–solid ratio and screw speed. Additionally, during roller compaction, the density and porosity of intermediate ribbons are controlled and monitored using PAT by controlling process parameters, such as roller compaction; roller speed, pressure and spacing; screw feed rate; vacuum degassing pressure, and mill speed [217,218]. The research that performed quality control by controlling IQAs and CPPs through monitoring using a PAT tool in granulation process is presented in Table 3.

#### 4.2.2. Application of PAT in Granulation Process for CPV

- High-shear granulation

Huang et al. measured the size and number of particles in real-time using an in-line monitoring method in a high-shear granulator using FBRM C35 [31]. Jiménez et al. performed monitoring of moisture content using NIRS in a high-shear mixer. After applying the LOD data as a reference method, a multivariate correction model by PLS was developed to show high precision. As the moisture content increased, the baseline of the spectrum shifted upward, indicating an increase in granule size [219]. Reddy et al. used Raman spectroscopy to control the moisture content, temperature, and wetting of the intermediate product using on-line monitoring. A calibration model was formed using PLS as a multivariate tool. As a result, it was confirmed that the complexity of changing the shape of particles depending on the CPP affects drug dissolution during processing. This indicates that the monitoring of wet granules is an essential factor in the clinical stage, and controlling it using a PAT tool is an efficient quality-control method [8]. Shikata et al., during wet granulation, used NIRS to perform the in-line monitoring of granulation properties, such as particle size, density, and fluidity. To avoid contamination of the probe and to maintain sample homogeneity, a non-invasive monitoring method was developed in the presence of compressed air, and spectra were collected in the range of 1100–2150 nm. The data spectrum was preprocessed via SNV and was used to determine the endpoint of the granulation process by understanding the correlation between CPPs and IQAs via PCA. They showed that granule properties could be predicted by forming qualitative and quantitative calibration models using PCA and PLS [220]. Hansuld et al. used AAE as a PAT tool to measure density and particle size distribution through the on-line monitoring of particle property changes during wet granulation. After the blending process using the high-shear granulator, process monitoring was performed by controlling parameters such as impeller speed, binder amount, and binder spray rate through DoE. PLS and PCA were used as multivariate statistical tools. The authors assessed the correlation among the impeller speed, the binder amount, and the endpoint by confirming the PCA modeling result. Moreover, they demonstrated that monitoring through AAE could detect the endpoints of the granulation process, suggesting that drug development time could be shortened by controlling the process and controlling variability more easily [221].

- Fluidized-bed granulation

Roßteuscher-Carl et al. [222] and Reimers et al. [60] used an in-line SFV to monitor the particle-size distribution during fluid-bed granulation. The sapphire window of an SFV probe uses an internal compressed-air-supply system to prevent window contamination. After the SFV data is corrected through modeling and preprocessing, data evaluation is performed to verify the accuracy of monitoring. Factors such as the probe’s position and the binder spray speed must be properly controlled in the fluid-bed granulation process. Alcala et al., Kona et al., and Gavanet et al. measured moisture content by monitoring using an NIRS probe connected to a fluidized bed [223,224,225]. Spectrum data collected in real-time were preprocessed with SNV, MSC, first derivative, etc., and then analyzed with multivariate statistical tools, such as PCA, PLS, and MLR. The reference value was LOD, and after correcting and forming the prediction model, data evaluation through RMSEC and RMSEP proves the predictive ability and usability of NIRS. Rantanen et al. evaluated the granules’ moisture content and bulk-density characteristics by controlling the change of the inlet air condition through monitoring using NIRS during the granulation process [146]. In addition, it was confirmed that IQAs such as residual content and granule flowability of intermediate products affect hardness and weight, which are CQAs of finished products. The collected data were corrected through PLS modeling, and high accuracy was verified through low prediction errors. In other words, it shows that quality control can be continuously performed by introducing a PAT framework into the fluid-bed granulation process and controlling various IQAs and CPPs through real time monitoring.

- Twin-screw granulation

Meng et al. used NIRS, Raman spectroscopy, and 3D high-speed imaging cameras during twin- screw granulation to evaluate the size and shape changes, physical properties, and content of the granules. Granule size and shape were monitored in real time using Eyecon 3D imaging, and granule properties, such as granule size, porosity, density, and flowability, were quantitatively predicted using NIRS. Moreover, Raman spectroscopy was used to evaluate the uniformity of the drug content and the solid-state transformation of the granules. This study shows that the PAT tool can predict granule properties with high accuracy and precision under various operating conditions [149]. Harting et al. used a Raman RXN2 hybrid analyzer to measure changes in drug content during the split feeding process. UV-VIS spectroscopy was used as a reference method for PLS modeling. The authors confirmed that the predictive ability was high using the low RMSEP value, indicating that the blending process was performing properly [9,226].

- Roller-compaction granulation

**Figure 2:**
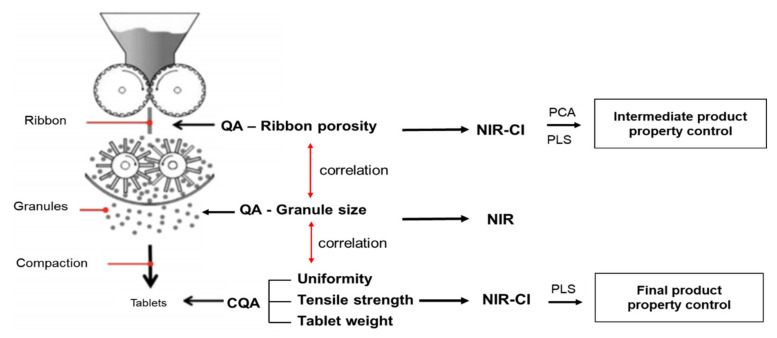
Overview of the process monitoring of roll compaction and tableting: the implementation of near-infrared chemical imaging (NIR-CI) to obtain information related to the physical or chemical properties of intermediate products or finished products [175]. Reproduced with permission from Milad Khorasani, European Journal of Pharmaceutics and Biopharmaceutics; published by Elsevier, 2015.

Acevedo et al. monitored the ribbon density using NIRS on a roller compactor. During the roller-compaction process, the PCA model developed based on the spectrum obtained by in-line monitoring shows that the physical change of the ribbon can be detected and analyzed qualitatively [174]. The high R^2^ value of the calibration model obtained using PCA and PLSR shows the accuracy of quality prediction through the PAT. Therefore, using this approach, it is possible to measure the IQAs of ribbons and granules in the roller-compaction process and the CQAs produced in the tableting process, as shown in Figure 2. In the study, the authors demonstrate the potential utility of PAT in controlling continuously connected manufacturing process lines [175]. In addition, NIRS is used to monitor the IQAs of intermediate products: ribbon density, tensile strength, and drug content. Each spectrum collected was preprocessed by MSC followed by PLS for multivariate data analysis. The calibration model was set up using reference methods, such as LOD. The low calibration error confirmed that the process monitoring using NIRS during the roller compaction process is suitable [176]. Gupta et al. monitored ribbon density and drug content using NIRS and microwave sensors in the roller compaction process [227]. Khorasani et al. performed process monitoring using NIR and NIR-CI in a continuous roller compression granulation process. DoE was performed through factorial design using the roller pressure and roller speed selected as CPPs, and experiments were conducted on 15 test points. As a result, it was found that the lower porosity of ribbon produced through the roller compactor, the larger the particle size of the granules produced by the milling of the ribbon. It was confirmed that it adversely affects the weight of the tablet. Therefore, in the roller-compaction process, the porosity of the ribbon was monitored using NIRS, and the change in the granule-size profile after milling was qualitatively monitored using on-line NIRS. In this literature, through process monitoring using NIR and NIR-CI as a PAT tool, the IQAs of intermediate ribbons can be measured by controlling the roller pressure and roller speed in the roller-compression process, and the IQAs of granules can be thoroughly managed. It also demonstrates the applicability of PAT as a quality tool and predictive tool in the continuous manufacturing of the pharmaceutical industry [175].

**Table 3:** Summary of the PAT-based modeling method applied in the granulation process.

Equipment Type	Monitored IQAs in Process	PAT Tool (Model)	Related CQAs of Finished Product	Ref
High shear mixer wet-granulator	- Moisture content	MEMS-FPI NIR spectrometer (N-Series 2.2, Otaniemi, Espoo, Finland)	- Moisture content - Dissolution	[228]
	- Moisture content - Median granule size - Granule-size distribution	Acoustic emission (Physical Acoustics, Princeton Junction, NJ, USA)	- Moisture content - Dissolution - Content uniformity	[229]
	- Bulk density - Moisture content - Granule-size distribution - Porosity	FT-NIR spectrometer (ABB Bomem MB-160, Q-Interline, Roskilde, Denmark)	- Moisture content - Dissolution - Content uniformity - Tablet hardness	[230]
	- Density - Granule-size distribution	ICP sensor signal conditioner (PCB Piezotronics)	- Dissolution - Content uniformity	[221]
	- Density - Granule-size distribution	Audible acoustic emissions, (PCB Piezotronics condenser microphones model 130D20)	- Dissolution - Content uniformity	[181]
	- Moisture content - Granule-size distribution	Fourier transform (FT)-NIR spectrometer (Bruker Optics Vector 22/N, Billerica, MA, USA)	- Moisture content - Dissolution - Content uniformity	[219]
	- Density - Moisture content - Granule size	AOTF-NIR spectrometer (Luminar 4030 NIRS, Brimrose, Sparks, MD, USA)	- Moisture content - Dissolution - Content uniformity	[220]
	- Granule formulation - Moisture content	Raman, RXN1-PhAT probe system (Kaiser Optical Systems, Inc., Ann Arbor, MI, USA) Antaris NIR spectrometer (Thermo Fisher, UK)	- Moisture content - Dissolution - Content uniformity	[8]
	- Drug content - Moisture content	MPA Multi Purpose FT-NIR Analyzer (Bruker-Optics, Billerica, MA, USA)	- Moisture content - Dissolution - Content uniformity - Assay	[231]
	- Granule-size distribution	FBRM C35	- Dissolution - Content uniformity	[31]
	- Drug content	NIR Spectrometer	- Dissolution - Content uniformity - Assay	[232]
Fluidized-bed granulator	- Moisture content - Granule size	SFT probe (Parsum IPP 70; Gesellschaft für Partikel-, Strömungs-, und Umweltmesstechnik GmbH, Chemnitz, Germany)	- Moisture content - Dissolution - Content uniformity	[233]
	- Granule size - Granule-size distribution	Parsum IPP 70 (Gesellschaft für Partikel-, Strömungs- und Umweltmesstechnik, Chemnitz, Germany)	- Dissolution - Content uniformity	[234]
	- Bulk density - Moisture content - Granule-size distribution	QualitySpec ASD NIR spectrometer	- Moisture content - Dissolution - Content uniformity	[223]
	- Moisture content - Granule density - Granule size	Microwave resonance technology Hydorpharm fbma (Döscher & Döscher GmbH, Hamburg, Germany)	- Moisture content - Dissolution - Content uniformity	[235]
	- Moisture content	FOSS NIRSystems XDS Process Analyzer (FOSS NIRSystems, Inc., Laurel, MD, USA)	- Moisture content - Dissolution	[224]
	- Moisture content	MicroNIR PAT-U spectrometer (Viavi Solution, San Jose, CA, USA)	- Moisture content - Dissolution	[225]
	- Moisture content	FT-NIR spectrometer (Buhler NIRVIS, Uzwil, Switzerland)	- Moisture content - Dissolution	[236]
	- Granule-size distribution	Spatial filter velocimetry probe (Parsum IPP 70; Gesellschaft für Partikel-, Strömungs- und Umweltmesstechnik, Chemnitz, Germany)	- Dissolution - Content uniformity	[237]
Twin-screw granulator	- Granule size - Solid state	RamanRxn1 spectrometer (Kaiser Optical Systems, Ann Arbor, MI, Michigan) Spatial Filter Velocimetry probe (Parsum, Chemnitz, Germany) Fourier-transform NIR spectrometer (Thermo Fisher Scientific, Zellik, Belgium, Nicolet Antaris II near-IR analyzer)	- Dissolution - Content uniformity - Physical attributes - Degradation	[147]
	- Moisture content - Granule-size distribution	NIR chemical imaging system (SWIR, Specim Ltd., Oulu, Finland)	- Dissolution - Content uniformity - Moisture content	[72]
	- Drug content - Granule size	Raman RXN2 hybrid analyzer (Kaiser Optical Systems, Ann Arbor, MI, USA)	- Dissolution - Content uniformity - Assay	[226]
	- Granule-shape variation - Granule-size distribution	MATRIX-F emission FT-NIR spectrometer (Bruker Optics, Billerica, MA, USA) Eyecon (Innopharma Laboratories, Dublin, Ireland) Kaiser PhAT System analyzer Raman (Kaiser Optical Systems, Ann Arbor, MI, USA)	- Dissolution - Content uniformity	[149]
	- Moisture content - Granule-size distribution	SentroProbe DR LS NIR	- Dissolution - Content uniformity - Moisture content	[131]
	- Moisture content - Granule size	Raman RXN2 hybrid analyzer (Kaiser Optical Systems, Ann Arbor, MI, USA)	- Dissolution - Content uniformity - Moisture content	[9]
	- Drug content - Moisture content	Hyperspectral camera (SWIR, Specim Ltd., Oulu, Finland)	- Dissolution - Content uniformity - Moisture content - Assay	[238]
Roller compactor	- Density - Moisture content - Granule-size distribution - Tensile strength	Near-infrared spectrograph (Control Development, Inc., South Bend, IN, USA)	- Dissolution - Content uniformity - Moisture content - Tablet hardness	[239]
	- Drug content - Moisture content - Relative density - Tensile strength - Ribbon density	Near-infrared spectrograph (Control Development, Inc., South Bend, USA)	- Dissolution - Content uniformity - Moisture content - Tablet hardness - Assay	[176]
	- - Ribbon density	NIR spectrograph	- Dissolution - Content uniformity	[240]
	- Ribbon density	CDI non-contact diffuse reflectance spectrometer (SNIR 278, Control Development Inc. South Bend, IN, USA)	- Dissolution - Content uniformity	[174]
	- Drug content - Density - Ribbon porosity - Tensile strength	Near-infrared (NIR) spectrometer (Control Development, Inc., South Bend, IN, USA)	- Dissolution - Assay - Content uniformity - Tablet hardness	[241]
	- Drug content - Moisture content - Ribbon density	Turbido OFS12S- 120H NIR, microwave sensor (Solvias AG, Basel, Switzerland) microwave sensor	- Dissolution - Assay - Content uniformity - Moisture content	[227]
	- Granule-size distribution - Ribbon porosity - Tablet tensile strength	NIR-chemical images spectrometer (Headwall Photonics model 1002A-00371, FOSS A/S, Hillerod, Denmark)	- Dissolution - Content uniformity - Tablet hardness	[175]
	- Granule size - Ribbon porosity	Fourier-transform NIR spectrometer (ABB Inc., Quebec, QC, Canada) NIR chemical imaging system (Headwall Photonics model 1002A-00371, FOSS A/S, Hillerod, Denmark)	- Dissolution - Content uniformity	[242]
	- Moisture content - Ribbon density	167PA-0501-01000 forked microwave sensor (Sartorius Stedim in Bohemia, New York, NY, USA) Turbido OFS-12S120H NIR sensor (Solvias AG in Basel, Switzerland)	- Dissolution - Content uniformity - Moisture content	[243]

### 4.3. Drying Process

The drying process for a solid dosage form is usually done simultaneously with the granulation process or after the granulation process to evaporate the sprayed water. Drying is used to assure the long-term preservation of the product because it provides a dried drug product that quickly and completely rehydrates when added to the solvent [178]. One of the drying processes, the freeze-drying process, may be performed depending on the needs of the formulation. Freeze drying is primarily used in the pharmaceutical manufacturing process to preserve the initial properties of the raw products after dehydration [18]. In contrast to the drying process that uses high temperatures, freeze-drying is widely used at lower operating temperatures to reduce damage to drug properties [244]. In the drying process, there is a need to achieve CPV by monitoring the drying temperature, drying rate, and chamber pressure, which are process parameters that affect CQAs in real-time. A PAT is primarily used during the freeze-drying process based on product temperature, chamber pressure, and sublimation flux estimates, which affect critical quality characteristics [158]. However, this can lead to process failure because it is challenging to grasp various process variations, including failure, due to unexpected changes in the set value of variables or the fluid mechanics of the chamber.

#### 4.3.1. Monitored IQAs in the Drying Process

The drying process should introduce real-time PAT tools, such as spectroscopy and imaging systems, to quickly provide information on CQAs, including water-to-ice conversion, product crystallization, solid characterization, residual moisture determination, and protein integrity [131].

#### 4.3.2. Application of the PAT Framework in the Drying Process for CPV

**Figure 3:**
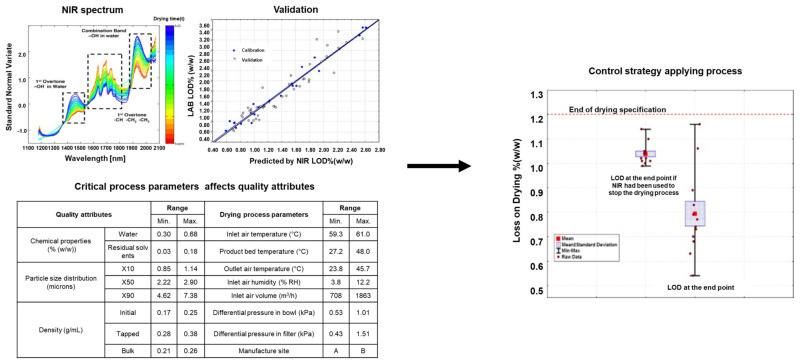
Positioning of a near-infrared (NIR) spectrometer in a fluidized-bed drying process and the steps of the process control strategy using PAT [248]. Reproduced with permission from Antonio Peinado, Journal of Pharmaceutical and Biomedical Analysis; published by Elsevier, 2011.

The research that performed quality control by controlling IQAs and CPPs through monitoring using a PAT tool in drying process is presented in Table 4. In a series of reports, Brülls et al. used Raman spectroscopy and NIRS for the in-line process monitoring of the solid state of mannitol, an endpoint of the crystallization process during freeze-drying. Mannitol was crystallized, but its solid state did not change during freeze-drying. Multivariate data analysis was performed using PCA and/or multivariate curve resolution and by comparing the reference spectra of mannitol forms [6]. Brouckaert et al. reported continuous NIR-CI as a PAT tool during freeze-drying. A PLSR model was used in multivariate analysis for quantifying the moisture content in chemical images and analyzing inhomogeneity in moisture content by PCA. Moreover, the authors evaluated NIR-CI as a PAT tool for the at-line monitoring of the freeze-drying process. The solid state of mannitol samples was pictured by a CI system. The collected chemical images varied after corresponding analysis by PCA to detect inhomogeneity in the moisture content. Furthermore, a PLSR model, with each pixel of the chemical images presented, was constructed for the quantification of the moisture content [245]. Bosca et al. used a soft sensor as a PAT tool to estimate the residual ice content and values of heat coefficients of the vial in the primary drying stage during the freeze-drying process [246]. In-line monitoring was performed to confirm whether the product temperature was maintained below the limit value in the entire batch. The soft sensor wireless measurement system showed that it can be used in industrial-scale freeze-dryers. Moreover, there is an example of applying PAT to a drying process using a fluidized-bed dryer instead of a freeze-dryer. Räsänen et al. performed in-line monitoring using NIRS for the stepwise dehydration phenomenon occurring during the fluidized-bed drying process. They showed that the process can be controlled by observing changes in the solid state that affect the stability of the solid and by grasping the effect of process variables for the production of the finished products [236]. Peters et al. selected NIRS and MRT as PAT tools for monitoring the moisture content of granules in real time during the drying process using a fluidized-bed dryer. They analyzed the obtained data by MLR and PLS as a quantitative calibration model, confirming the difference between the two instruments. Unlike with previous NIRS devices, monitoring large amounts of moisture by MRT was difficult, indicating that the NIRS equipment is suitable for continuous process monitoring and control [247]. Furthermore, Figure 3 shows that PAT using NIR could be utilized for a control strategy determining the endpoint of fluidized drying process [248].

### 4.4. Coating Process

The coating process is an essential process for a pharmaceutical product to perform its intended action. For example, it is used to protect drugs from gastric acids in general, mask odors or tastes, or prevent drug interactions or for the prolonged release of drugs. Most parts of the coating process are performed in pans or fluidized-bed devices [1,256]. In a continuous process, equipment and scale changes can significantly impact finished-product quality, including coating thickness [136] and drug content [257]. Therefore, CPV using a control strategy such as application PAT, which enables continuous real-time monitoring, is required. Coating quality is determined by convoluted interaction effects associated with the process parameters, material attributes, environmental conditions, equipment design, and others [1]. For example, the finished-product quality is determined by CPPs such as the spray speed, pan speed, or fan loading in this process, which affect CQAs [258].

#### 4.4.1. Monitored IQAs in the Coating Process

The amount, thickness, and homogeneity of the coating monitored in the coating process are IQAs that affect the rate of drug release. This can affect the quality and stability of the finished product [136]. Therefore, with respect to quantitative modeling, there is a need for standards and methods that can directly measure, in real-time, the related coating properties in an accurate and reliable method. At present, in the pharmaceutical industry, destructive methods, such as cutting, are most common for directly measuring coating thickness. However, there is a need for noninvasive PAT tools to be introduced to avoid additional variability and errors [179]. The research that performed quality control by controlling IQAs and CPPs through monitoring using a PAT tool in coating process is presented in Table 5.

#### 4.4.2. Application of the PAT Framework in the Coating Process for CPV

- Pan coating

**Figure 4:**
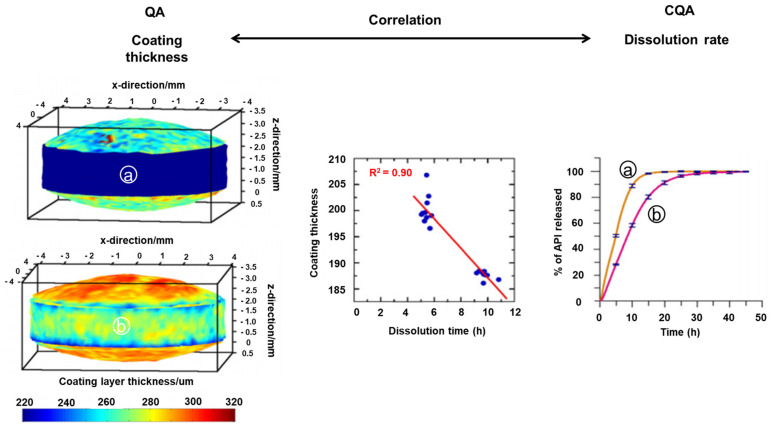
Schematic diagram showing the correlation between the coating thickness and the dissolution rate by analyzing the coating thickness of the tablet by applying a hyperspectral imaging system to the coating process [261]. Reproduced with permission from Louise Ho, Journal of Controlled Release; published by Elsevier, 2008.

PAT tools are used for the monitoring and process control of the coating process. Typically, tools include spectroscopic techniques, such as NIRS and Raman spectroscopy, as well as imaging techniques, including TPI [259]. El Hagrasy et al. used PAT tools, such as Raman spectroscopy, for monitoring the coating process. The intensity of the spectrum lines collected by this spectrometer increased in the coating material as the coating time increased. Raman spectral data processing was performed by relevant baseline correction, SNV, and second-order derivative preprocessing, while PLS was developed as a calibration model. Moreover, to build a calibration model of the correlation between the acquired spectrum and coating thickness values, the weight increase of the tablet over time was measured and used as a reference value. The results demonstrated that Raman spectroscopy can be used for quantifying coating uniformity [260]. Gendre et al. used NIRS in the real-time coating process to predict the increase in the mass and thickness of the coating material. In addition, they used TPI to regularly sample the coated tablets during the coating operation to determine the coating layer thickness [3]. Knop et al. showed that, when a terahertz wave is irradiated onto the coating surface of the tablet, it is reflected or partially penetrates the coating. The authors used in-line NIRS and TPI and analyzed the data using PLS calibration. Furthermore, preprocessed NIRS data were applied to the PCA and PLS calibration models for multivariate qualitative and quantitative analyses, respectively. Another reference method for quantifying tablet weight was used for building a calibration model from the in-line NIRS data. The calculated real-time near-infrared spectrum prediction was similar to the actual mass of the coating material measured by the reference method, i.e., weighing [256]. Ho et al. used TPI in coating analysis to predict the dissolution performance of sustained-release tablets. Furthermore, to evaluate coating quality, the thickness of the tablet-coating layer was selected to be measured parameters using TPI in this study. The authors found that TPI results correlate with the performance of the actual product and can predict the dissolution behavior of sustained-release tablets as shown in Figure 4 [261].

- Fluidized-bed coating

Hattori et al. studied a pharmaceutical manufacturing system based on the QbD concept of enteric-film-coated tablets, such as coating thickness, mass of coating, and hardness, which can be predicted through real-time monitoring using NIRS. By applying the PLS calibration model to predict tablet characteristics, the authors suggested that the polymer concentration of the coating suspension, which is a CPP, affects CQAs [4]. Andersson et al. used in-line NIRS in the pellet-coating process. The spectrometer was positioned in the fluidized-bed coater via a Wurster insert; PLS calibration was performed on 11 coating batches, and an evaluation was performed on eight additional batches. Comparing several data preprocessing methods, the best way to predict the coating thickness was by R^2^ = 0.97 [262]. Lee et al. investigated new approaches, including averaging and clustering, to develop rational dynamic correction models using the in-line spectra from randomly moving materials instead of offline spectra from fixed objects. The in-line spectrum collected in the dynamic coating process was not the same as that measured in the stationary tablet, indicating a significant difference from the offline spectrum. The prediction experiment presented a fairly good agreement between the measured average and predicted values [263]. Bogomolov et al. combined NIRS and Raman spectroscopy in the fluidized-bed pellet-coating process. Both data blocks, individually or in combination, were used to perform a PLSR analysis of the product’s moisture and the amount of sprayed coating material [264]. Lee et al. investigated pellet-coating thickness monitoring and its prediction using NIRS in the in-line fluidized-bed coating process. In addition, they used calibration models to predict the endpoints of a coating experiment [265]. Naidu et al. studied the application of NIRS in the Wurster coating process for monitoring cellulose content. They collected absorption regions for cellulose and the drug and correlated the absorption values by at-line monitoring and calibration using PLS. The predictive ability of the final validation model was evaluated by comparison with NIRS data [152]. Možina et al. proposed digital visual imaging for monitoring pellet-coating thickness, spherical diameter, and agglomeration in the fluidized-bed coating process. This is an imaging system comprising a black-and-white camera, lens, and LED lighting, which makes a number of measurements in a relatively short time and yields good statistical estimates; it controls the process by monitoring the average size increase of the pellets [266].

**Table 5:** Summary of PAT-based modeling method applied in coating process.

Equipment Type	Monitored IQAs in Process	PAT Tool (Model)	Related CQAs of Finished Product	Ref
Pan coater	- Drug content	PhAT System (Kaiser Optical Systems, Ann Arbor, MI, USA)	- Assay - Content uniformity	[172]
	- Coating thickness - Coating uniformity - Mass of coating	Photodiode array spectrometer (MCS 611 NIR 1.7H spectrometer, Carl Zeiss, Germany), TPI Imaga 2000 (TeraView Ltd., Cambridge, UK)	- Content uniformity - Dissolution	[3]
	- Drug release	Photodiode array spectrometer (MCS 611 NIR 1.7H spectrometer, Carl Zeiss, Germany)	- Dissolution	[267]
	- Coating growth - Moisture content	FT-NIR spectrometer (Matrix-F, Bruker Optik GmbH, Ettlingen Germany)	- Content uniformity - Moisture content	[257]
	- Coating endpoint	Raman RXN2 Analyzer (Kaiser Optical Systems, Ann Arbor, MI, USA)	- Content uniformity	[268]
	- Coating weight gain	Raman spectrometer (Kaiser Optical Inc., MI, USA), Raman microscope RAMANforce (Nanophoton Corporation, Osaka, Japan)	- Content uniformity	[269]
	- Coating thickness - Tablet color variability	Raman spectrometer with a 6 mm PhAT probe (RXN4 Analyzer, Kaiser Optical Systems, Ann Arbor, MI, USA), Unmodified computer scanner (Epson Perfection V800 Photo, Suwa, Japan)	- Content uniformity - Physical attributes (appearance)	[132]
	- Coating thickness	Raman RXN2 analyzer (Kaiser Optical Systems, Inc., Ann Arbor, MI, USA)	- Content uniformity	[270]
	- Coating thickness	NIR spectrometer (Foss NIRSystems, Inc., Laurel, MD, USA)	- Content uniformity	[271]
Fluidized- bed coater	- Coating thickness	FT-NIR spectrometer (FTPA 2000-260; ABB Bomem, Quebec, QC, Canada)	- Content uniformity	[263]
	- Coating thickness - Moisture content	RxN1 Raman spectrometer (Kaiser Optical Systems Sarl, Ecully, France) J&M TIDAS 1121 SSG Spectrometer (J&M Analytik AG, Essingen, Germany)	- Content uniformity - Moisture content	[264]
	- Coating thickness	FT-NIR spectrometer (FTPA 2000-260; ABB Bomem, Quebec, QC, Canada)	- Content uniformity	[265]
	- Drug content - Coating thickness - Coating weight	FOSS NIR Rapid Content Analyzer 5000 (FOSS NIR Systems, Inc., Laurel, MD, USA)	- Assay - Content uniformity	[151]
	- Coating thickness - Moisture content	NIR diffuse reflectance probe (Lighthouse probe, GEA Pharma Systems, Belgium)	- Content uniformity - Moisture content	[262]
	- Drug content	Thermoscientific Antaris-II FT-NIR process analyzer	- Assay - Content uniformity	[152]
	- Drug dissolution performance - Coating thickness	FBRM D600 (Mettler Toledo, Columbia, MD, USA) NIR ePAT611 (Expo Technologies LLC, Columbia, MD, USA) Pro-Raman Process Analyzer (TSI, former ENWAVE Optronics, Inc., CA, USA)	- Content uniformity - Dissolution	[153]

### 4.5. Tableting Process

In the tableting process, the compression pressure used in the tableting press is measured via an internal sensor as a major process parameter because it can have an enormous impact on some CQAs and finished-product properties. However, there are few commercial tools that can measure CQAs, such as tablet weight and hardness, in real-time monitoring. A destructive technique is usually used to determine the hardness and drug content, which are physical properties, of a tablet manufactured by blending API and excipients and compressing them into tablets. By contrast, NIRS, Raman spectroscopy, and chemical imaging are nondestructive techniques that provide the simultaneous measurement of chemical and physical properties during the tableting process. In this manner, the spectrum of compressed tablets, which is collected via spectroscopy or imaging, is affected by small changes in tablet and surface shape (such as rectangular or concave) and tablet hardness [272].

#### 4.5.1. Monitored IQAs in the Tableting Process

In general, the PAT tool applied in the tableting process is positioned on the powder-feed frame of the tableting press and principally used to monitor the blending uniformity and drug content and accordingly provide feedback control of the process. The research that performed quality control by controlling IQAs and CPPs through monitoring using a PAT tool in tableting process is presented in Table 6.

#### 4.5.2. Application of PAT Framework in Tableting Process for CPV

**Figure 5:**
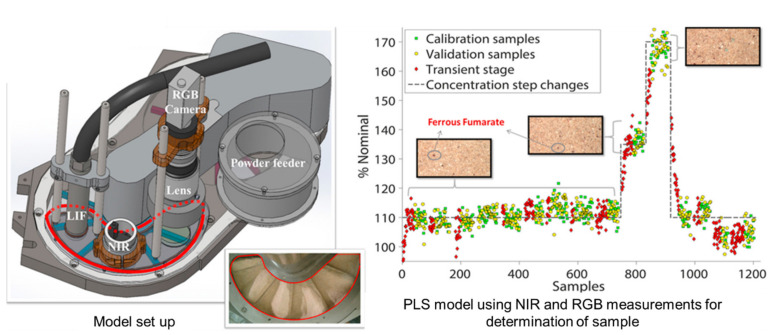
Schematic of the position of three process analytical technology (PAT) tools on the tablet-press feed-frame for monitoring experimental samples [277]. Reproduced with permission from Pedro Durão, Talanta; published by Elsevier, 2017.

Wahl et al. analyzed powder content uniformity using an NIRS probe mounted on the powder-feed frame in the tableting press. They used the DoE for the accurate prediction of powder composition. In addition, they measured the standard of drug content using a UV-VIS light spectrometer. Agreement was confirmed between the in-line NIRS data and UV-VIS data. The authors performed the PCA of the spectrum to check for major process deviations, such as excessive drug content in the powder [273]. Likewise, Dalvi et al. studied monitoring for API (ibuprofen) content in the tableting process. As previously suggested, the NIRS probe was installed on the powder-feed frame, and a UV-VIS spectrometer was used as a reference method. In-line monitoring, as well as offline monitoring, was performed using NIRS, and the in-line spectrum of the compressed tablet was quantitatively evaluated by PLS analysis. The PLS-predicted content of the tablet was in good agreement with the corresponding content obtained by the UV-VIS assay [272]. The Li et al. study is an example of PAT using Raman spectroscopy, another spectral technology tool in the tablet compression process. The authors positioned the Raman spectroscopy probe on the powder-feed frame of a tablet-compression system for the quantitative real-time assessment of a tableting press. The contact Raman spectroscopy probe was directly immersed into the powder. The authors developed and validated both in-line and offline calibration strategies to determine the blend content during tablet compression. PCR was applied on mean-centered data for the quantitative analysis of drug content in the mixture. The same spectral range, preprocessing spectra, and regression method were used for the offline, static, and in-line models, and HPLC was used as a reference method [274]. Chemical imaging can also be used as a nondestructive technology to determine tablet density profile in the tableting process [275]. Ellison et al. used an NIR-CI system for monitoring the tableting process. They monitored not only the physical properties of tablets but also the compression forces within a single tablet. Tablet surfaces and edge-absorbance images were acquired at a single wavelength for density analysis. They quantitatively evaluated the difference in compaction based on the collected images and density distribution. As a measure of density uniformity resulting from the tablet compression force, the ratio of the average absorbance was measured from the bottom to the top of the tablet surface. Considering the linear relationship between this absorbance ratio and the force-transmission ratio, the tablet-release force was also related to the increasing absorbance ratio, which means better force transmission and more uniform density [276]. Moreover, Gosselin et al. used PAT tools, such as light-induced fluorescence spectroscopy, NIRS, and color (RGB) imaging, to monitor the composition of powder flowing in the tableting process to determine the content of components. As shown in Figure 5, the three probes could be used to monitor powder flowing in real time and are suitable for detecting undesirable phenomena, including the feed-frame dynamics and separation [277].

**Table 6:** Summary of PAT-based modeling method applied in tableting process.

Equipment Type	Monitored IQAs in Process	PAT Tool (Model)	Related CQAs of Finished Product	Ref
Direct compression	- Tablet hardness	NIR spectrometer (NIR systems Rapid Content Analyzer Model 5000, Silver Spring, MD, USA)	- Content uniformity - Dissolution	[154]
	- Drug content	NIR spectrometer (VisioNIR ls, Uhlmann VisioTec GmbH, Laupheim, Germany)	- Assay - Content uniformity	[213]
	- Content uniformity	NIR spectrometer (FOSS NIRSystems Model 5000, Silver Spring, MD, USA)	- Content uniformity	[273]
	- Drug content	NIR spectrometer (MPA, Bruker Optics, Ettlingen, Germany)	- Assay - Content uniformity	[278]
Rotary tablet compression	- Drug content	NIR spectrometer (SentroPAT FO, Sentronic GmbH, Germany)	- Assay - Content uniformity	[279]
	- Drug content	NIR spectrometer (ePAT611, Expo Technology, St Louis, MO, USA)	- Assay - Content uniformity	[2]
	- Drug content	NIR spectrometer (SentroPAT FO, Sentronic GmbH, Germany)	- Assay - Content uniformity	[136]
	- Drug content - Content uniformity	NIR spectrometer (MCS 611 NIR 2.2, Carl Zeiss, Germany)	- Assay - Content uniformity	[280]
	- Content uniformity	NIR spectrometer (Foss NIRSystems, Inc., Laurel, MD, USA)	- Content uniformity	[281]

## 5. Conclusions

In the pharmaceutical industry, as the importance of pharmaceutical quality has increased, various strategies and methods have been introduced, such as CPV, QbD, and RTRT, for high-quality pharmaceuticals produced by continuous manufacturing. CPV refers to a monitoring and evaluation approach for process control and performance evaluation and is guaranteed through a system based on RTRT. At this time, the PAT tool can be usefully used as a strategy for process monitoring and control for RTRT. In addition, when integrated with the QbD approach, the PAT framework enables more efficient RTRT. The QbD approach identifies the correlations between IQAs, CPPs, and CQAs during the process and manages them appropriately during the process. The PAT tool is used to control real-time process monitoring. Because this minimizes resources such as time and cost for the production of high-quality pharmaceuticals and maximizes quality assurance, we believe that the PAT initiative in CPV can make a significant contribution to the scientific and technical aspects of the pharmaceutical industry. To demonstrate the usefulness of the PAT framework as a control strategy via RTRT, this review provides process monitoring using the PAT framework in the pharmaceutical industry’s solid-form manufacturing processes, such as blending, granulation, drying, tableting, and coating. As suggested in the paper, to establish this management strategy, the first step is to understand the correlation between CPPs and IQAs to increase the understanding of materials, processes, and process parameters and select the most suitable tool considering the characteristics of the PAT tool. After that, data can be collected by selecting a method of collecting data by applying the PAT tool to the process (at-line, on-line, in-line) and applying the PAT tool to the device for real-time monitoring. Depending on the tool, the collected data needs to be corrected, and a mathematical preprocessing technique must be used as a tool for this. Data analysis and modeling are performed using a chemometric model. Finally, by evaluating the data measured through monitoring, the accuracy of process monitoring using PAT in the process is verified. In other words, this literature shows that accurate and efficient process monitoring using the PAT framework is possible. It demonstrates that PAT can be used as an essential tool for process- and quality-control strategies in CPV during the pharmaceutical manufacturing process.

## Figures and Tables

**Table 1:** Effect of critical process parameters (CPPs) on intermediate quality attributes (IQAs) for the solid dosage form.

Process		Critical Process Parameter	Intermediate Quality Attributes	Justification	Ref
Blending		Blending time	- Drug content - Blending uniformity	If the blending time is long, separation may occur depending on the particle characteristics, which may affect the content and content uniformity of the mixture.	[25,26]
		Blending speed	- Blending uniformity	When blending above the optimum blending speed, the particles adhere to the wall of the blender by centrifugal force, which may affect the uniformity of the content of the mixture.	[25,26,27,28,29]
		Order of input	- Drug content - Blending uniformity	The order of input of additives has little effect on content and content uniformity because of the blending process in the blender. However, the effect of the input of the lubricant may affect the content and content uniformity.	[26]
		Environment	- Moisture content - Drug content	If temperature and humidity are not controlled, it may affect the moisture content of the mixture, and the content and content uniformity may be affected depending on the moisture and thermal stability of the drug.	[26]
		Filling level	- Drug content	Since the charging rate affects the movement of the particles, it can cause blending non-uniformity. This can affect the content and content uniformity of the mixture.	[25,26,27]
Granulation	High-shear granulation	Binder solvent amount	- Granule-size distribution - Granule strength - Flowability	When the amount of liquid increases, the powder is completely wetted, which impedes the particle flow in the granulator, which can affect the particle-size distribution of the granules by increasing the residence time and torque value. When the amount of liquid is insufficient, weak granules are formed.	[30,31]
		Binder solvent concentration	- Bulk/apparent/true density - Granule-size distribution	The concentration of the binding liquid has a direct relationship with the binding force and can affect the density and particle-size distribution of the granules.	[32,33,34]
		Binder solvent spray rate	- Drug content - Granule size - Granule strength	The binder solvent spray rate is directly connected to the size of the granules. If it is too slow, the process time is lengthened, and it is difficult to form granules; if it is too fast, a mass may be formed. Therefore, it can affect the granule-size distribution and density.	[35,36,37,38]
		Filling level	- Drug content	The filling level affects the movement of particles in the granulator ball, so that fine granules may be generated due to an increase in the number of collisions between the granules and an increase in strength. This can affect the content and uniformity of the granules.	[39,40]
		Impeller speed	- Granule density - Flowability - Granule strength - Bulk/apparent/true density - Granule-size distribution	The speed of the impeller determines the state of the granules. Accordingly, the porosity and density of the granules may be affected, and the particle-size distribution and flowability of the granules may be affected. In addition, as the impeller speed increases, it may affect the granule growth due to coalescence, so it may affect the granule size.	[30,35,41,42,43,44,45]
		Chopper speed	- Granule-size distribution - Bulk/apparent/true density - Flowability	Since the chopper speed plays a role in breaking the mass of granules, it can affect the density of the granules, the particle-size distribution, and the flowability of the granules.	[30,37,41,46]
		Massing time	- Granule-size distribution - Granule strength - Drug content uniformity - Bulk/apparent/true density - Flowability	The massing time is a factor that determines the main physical properties of the granules. Depending on the massing time, the strength of the granules and the density of the granules can be affected, and thus, the flowability and particle-size distribution can also be affected. Excessive massing time can result in granule growth by coalescence, which can affect granule size. Accordingly, it may affect the content uniformity of the granules, which may affect formation of granules.	[31,36,41,47,48,49]
		Mill screen size	- Granule-size distribution - Flowability - Bulk/apparent/true density	The mill screen size can affect the physical properties of the granules, such as the density and flowability of the granules, due to a large correlation with the particle-size distribution of the granules.	[35]
		Nozzle type	- Granule size - Granule-size distribution - Flowability	The nozzle position affects the spray angle of the binder solvent, which can affect the agglomeration and growth of the granules, but the effect is negligible. In addition, the size of the nozzle hole affects the distribution of the binder solution. However, this has little effect when adjusted with other process variables.	[39,50]
	Fluidized-bed granulation	Binder amount	- Granule-size distribution - Flowability	When the amount of liquid increases, the powder is completely wetted, which impedes the particle flow in the granulator, which can affect the particle-size distribution of the granules by increasing the residence time and torque value. When the amount of liquid is insufficient, weak granules are formed.	[51]
		Binder concentration	- Bulk/apparent/true density - Granule-size distribution	The concentration of the binding liquid has a direct relationship with the binding force and can affect the density and particle-size distribution of the granules.	[52,53,54,55,56,57]
		Binder spray rate	- Bulk/apparent/true density - Granule size - Granule-size distribution	The binder solvent spray rate is directly connected to the size of the granules. If it is too slow, the process time is lengthened and it is difficult to form granules; if it is too fast, a mass may be formed. Therefore, it can affect the granule-size distribution and density.	[53,54,55,56,57,58,59,60]
		Air volume/temperature/humidity	- Bulk/apparent/true density - Granule-size distribution - Flowability	Higher temperature increases fineness due to rapid drying, and lower temperature causes granules to agglomerate, resulting in harder and larger granules. This can affect the density, flowability and particle-size distribution of the granules. The flow of particles is determined according to the air-supply flow rate, and if it is too high, the degree of blending due to process loss may be lowered, which may affect the density, flowability, and particle-size distribution of the granules. The air-supply humidity determines the size of the granules, which can affect the particle-size distribution of the granules.	[52,53,59,61]
		Nozzle position	- Granule size - Granule-size distribution	The position of the nozzle affects the spray angle of the binder solvent, which can affect the agglomeration and growth of the granules, but the effect is negligible.	[54]
		Nozzle type	- Bulk/apparent/true density - Granule-size distribution	The nozzle type affects the way the binder is sprayed into the fluidized-bed of the particles, which can affect the particle-size distribution or density of the granules.	[54,62]
		Drying temperature/time	- Granule-size distribution - Flowability - Granule density - Moisture content	It can be determined according to the heat and moisture stability of the drug. If the drying time is short or the granules are not sufficiently dried due to the low drying temperature, the moisture content of the granules may be affected. If it is too high, fines may occur due to over-drying, which may affect the flowability and density of the particles.	[59,61]
		Environment	- Moisture content - Drug content	If the temperature and humidity are not managed, it may affect the moisture content of the granules, and the moisture and thermal stability of the drug may affect the content and content uniformity of the granules.	[59,63]
	Twin-screw granulation	Binder viscosity	- Granule-size distribution	When the binder solvent viscosity is high, there is a risk of granule mass, which may affect the size and particle-size distribution of the granules.	[64]
		Liquid to solid ratio	- Granule-size distribution - Flowability	If the amount of liquid inside the granulator increases, the powder may become excessively moistened and impede the flow of the inside. This increases the residence time and can thus affect the size and particle-size distribution of the granules.	[65,66,67,68,69]
		Feeder rate	- Bulk/apparent/true density - Granule-size distribution - Flowability	The feed rate of the powder affects the residence time, and due to the low feed rate, the inside of the granulator is not completely filled, and the residence time may be lengthened. This can affect granule properties, such as the particle-size distribution, density and flowability of the granules.	[65,66,67,70]
		Screw speed	- Density - Granule-size distribution - Ribbon uniformity	The screw speed can affect the residence time and, accordingly, the particle-size distribution and density of the granules.	[63,65,66,67,71,72,73,74]
		Screw type	- Density - Granule-size distribution	The type of screw is affected by the shape and angle of the screw to be engaged or the kneading pattern of the kneader part. This affects the amount of filling inside the granulator and can directly affect the compression and crushing of agglomerated particles and the distribution of the granules.	[65,66,69,75]
		Filling level	- Granule-size distribution - Bulk/apparent/true density	The feeder amount is directly related to the residence time and can affect the particle-size distribution and density of the granules.	[65,71]
		Residence time	- Granule size - Granule-size distribution	The residence time of the powder can affect the size and particle-size distribution of the granules.	[66,72,75]
	Roller compaction	Roller compactor type	- Ribbon density - Granule-size distribution - Flowability	Depending on the type of roller compactor, the principle of operation is different, which can affect the properties of the ribbon and the powdery properties of granules (roller width, roller diameter). The larger the diameter of the roller, the larger the compression area, so it may affect the characteristics of the ribbon, but, in general, the diameter of the roller is used as a fixed factor, so the effect on the intermediate product is insignificant.	[76]
		Roller pressure	- Drug content - Granule-size distribution - Flowability	Since the roller pressure determines the bonding force of the powder, it is judged to be directly related to the density of the ribbon. This may affect granule particle-size distribution, flowability and content uniformity after mill screening.	[35,76,77,78,79,80]
		Roller speed	- Ribbon density - Drug content - Granule-size distribution - Flowability	The roller speed is controlled by the screw speed, and it is judged that it has a direct relationship with the density of the ribbon as well as controlling the speed of the process. This affects the powder properties of the granules, which can affect the particle-size distribution and flowability of the granules.	[35,78,80,81,82,83,84]
		Roller gap	- Ribbon density - Granule density - Granule-size distribution	The roller gap affects the bonding force of the powder fed into the feeder, and may affect the ribbon density. This affects the powder properties of the granules after mill screening, which may affect the particle-size distribution and flowability of the granules. As the width of the roller changes, it is directly related to the maximum pressure of the roller, which can affect the density of the ribbon and thus the density and particle-size distribution of the granules.	[35,76,78,79,81,83]
		Feeder rate	- Ribbon Density - Granule-size distribution - Flowability	Input speed is directly related to roller pressure or roller spacing, which can affect the ribbon density, particle-size distribution and flowability of the granules.	[79,82]
		Feed screw speed	- Ribbon uniformity	Feed screw speed is a variable that is affected by roller pressure and roller spacing, and the effect is negligible.	[80]
		Residence time	- Ribbon uniformity	The residence time of the powder can affect the size and particle-size distribution of the granules.	[85,86,87]
		Mill screen size	- Granule-size distribution - Flowability	The size of the granulator can affect the physical properties of the granules, such as the density and flowability of the granules, due to a large correlation with the particle-size distribution of the granules.	[76,78,88]
		Mill speed	- Granule-size distribution - Flowability	The speed of the granulator can affect the powdery properties of the granules, but the effect is insignificant.	
Drying process		Drying time	- Particle size - Particle distribution - Drug polymorphic form - Moisture content - Bulk/apparent/true density	If the drying time is short, and the result is not fully dried, the moisture content may be affected. If the drying takes too long, fine powder may be generated due to over-drying, which may affect the flowability and distribution of the particles.	[89,90,91]
		Drying temperature		If the drying temperature is low, and the result is not fully dried, the moisture content may be affected. If the drying temperature is too high, fine powder may be generated due to over-drying, which may affect the flowability and particle distribution of the particles.	
		Inlet air temperature	- Moisture content	The thermal charge of the inlet drying gas reflects its capacity to dry the humid atomized droplets, and, therefore, higher inlet temperatures enable higher solvent evaporation rates.	[92]
		Air flow rate	- Particle distribution - Bulk/apparent/true density - Moisture content	The flow of particles is determined according to the air-supply flow-rate, and the air-supply flow-rate determines the size of the granules. This can affect the density and particle-size distribution. In addition, an increase in the air flow rate causes a higher evaporation rate.	[89,93]
Coating process		Rotation speed	- Coating uniformity	As the speed increases, the tablets apparently tumble through the spray zone rather than sliding flat, so the end exposure is more frequent, and the coating becomes more uniform.	[94,95,96]
		Nozzle diameter	- Coating thickness - Weight gain - Moisture content	The size of the sprayed droplet varies depending on the nozzle diameter. Therefore, since the amount of the coating liquid to be sprayed varies, this affects the moisture content and residual solvent.	[97,98]
		Inlet air temperature	- Coating uniformity - Moisture content	If the inlet air temperature is high, the tablets are excessively dried, and the surface becomes rough. If the inlet air temperature is low, the tablets stick together, and the moisture content of the tablets increases. Moisture content and coating uniformity are highly dependent on the incoming air temperature.	[99]
		Air flow rate	- Coating efficacy	The air flow rate prevents the sprayed coating solution from reaching the tablet. The faster the air flow, the lower the velocity of the sprayed droplet and the smaller the droplet size. Therefore, it affects the coating efficiency.	[100]
		Air volume	- Coating efficacy	An improper air layer due to worn or uneven drying may cause agglomeration between particles. An increase in air volume causes a decrease in spray density because the spray area increases as the droplet size decreases at the center of the spray.	[101]
		Coating solution composition	- Coated drug appearance - Coating uniformity - Hardness - Moisture content	In the case of functional coatings, the coating solution must contain an appropriate composition to deliver the desired effect of the drug, which affects the efficacy of the finished product. In addition, if the ratio of solids constituting the coating solution is high, efficient spraying becomes difficult, thus affecting the coating efficiency.	[102,103]
		Spray rate	- Coating uniformity	Too high a spray rate cause inadequate drying, twining, and sticking. Therefore, spray rate will have a significant impact on surface roughness and weight gain, thus affecting the coating uniformity.	[96,99]
		Atomizing air pressure	- Coating efficiency	Too high a spray pressure can lead to spray drying, and too low can cause agglomeration, which can have a significant impact on coating uniformity.	[104,105,106]
		Curing temperature/ time	- Coating efficiency - Moisture content - Hardness	The incorrect setting of the curing temperature and curing time will result in incomplete film formation. Thus, full film formation occurs when exposed to a certain curing temperature. The proper setting of curing time is necessary to achieve complete film adhesion.	[107,108,109]
Tableting process		Feeder speed	- Tablet porosity/density/solid fraction - Drug content - Weight variation	Low feeder speeds can lead to improper die filling, which can lead to weight changes and changes in hardness and thickness. Fast feeder speeds can overfill the die cavity and lead to weight variations and hardness and thickness variations.	[110]
		Rotary speed	- Drug content - Hardness - Weight variation	Rotary speed affects compressibility and even affects weight variation, which can affect drug content. A high rotary speed causes a much wider distribution of lubrication extent compared to the results from a low rotary speed. This may induce greater variability in hardness between tablets.	[111]
		Precompression force	- Tablet appearance - Thickness/dimensions - Tablet porosity/density/solid fraction - Hardness	Increasing compression force causes difficult particle rearrangement, deformation and fragmentation. Compression force affect tablet porosity, hardness, and density. In addition, depending on the tablet porosity, the degree to which moisture permeates into the tablet varies.	[112,113,114,115,116]
		Main compression force			
		Dwell time	- Weight variation	If the pressure holding time is too long, it deviates from the feeder speed, and inconsistent granules are filled into the die, which may cause weight fluctuations and affect the bonding force of the granules.	[110,111,117,118,119]
		Ejection force	- Tablet defects	The optimal compression force must be determined to obtain the desired tablet hardness	[120]

**Table 2:** Summary of PAT-based modeling method applied in blending process.

Monitored IQAs in Process	PAT Tool (Model)	Related CQAs of Finished Product	Ref
Blending uniformity	NIR Systems Model 5000 monochromator (Foss NIR Systems, Silver Spring, MD, USA) InSb focal plane array camera (IRC 160, LN_2_ cooled Cincinnati Electronics)	- Content uniformity - Dissolution	[205]
Drug content	NIR spectrometer (Control Development Inc., South Bend, IN, USA)	- Content uniformity - Dissolution - Assay	[21]
Blending uniformity	NIR spectrometer (Corona, type: remote NIR-HR, Carl Zeiss, Germany)	- Content uniformity - Dissolution	[206]
Blending uniformity	NIR spectroscopy Quantum 1200 Plus grating-based spectrometer (LT Industries, Rockville, MD, USA)	- Content uniformity - Dissolution	[207]
- Drug content - Blending uniformity - Tablet content uniformity	Kaiser RamanRxn2 hybrid in situ analyzer (Kaiser Optical Systems, Ann Arbor, MI, USA)	- Content uniformity - Dissolution - Assay	[168]
Blending uniformity	NIR Systems 6500 monochromator (NIR Systems, Silver Spring, MD, USA)	- Content uniformity - Dissolution	[208]
Blending uniformity	NIR spectrometer (NIR128L-1.7T-USB, Control Development, South Bend, IN, USA)	- Content uniformity - Dissolution	[209]
Blending uniformity	NIR spectral acquisition (MCS 611 NIR 2.2 spectral sensor, Carl Zeiss, Germany)	- Content uniformity - Dissolution	[136]
Blending uniformity	NIR spectrometer (LANCIR II spectrometer, BRUKER OPTIK GmbH, Ettlingen, Germany)	- Content uniformity - Dissolution	[23]
Blending uniformity	RamanRxn1 spectrometer (Kaiser Optical Systems, Ann Arbor, MI, USA), FT-NIR spectrometer (Bruker Optics, Belgium)	- Content uniformity - Dissolution	[27]
- Blending uniformity - Particle size changes	NIR spectrometer (Zeiss Corona 45, Carl Zeiss, Heidenheim, Germany)	- Content uniformity - Dissolution	[210]
Blending uniformity	FT-NIR-spectrometer (Spectrum 400, PerkinElmer, Waltham, MA, USA)	- Content uniformity - Dissolution	[211]
Blending uniformity	NIRFlex N-400 FT-NIR spectrometer (Büchi, Flawil, Switzerland)	- Content uniformity - Dissolution	[212]
Drug content	NIR spectrometer (SentroPAT FO spectrometer, Sentronic GmbH, Dresden, Germany)	- Content uniformity - Dissolution - Assay	[204]
Drug content	NIR spectrometer (VisioNIR ls, Uhlmann VisioTec GmbH, Laupheim, Germany)	- Content uniformity - Dissolution - Assay	[213]

**Table 4:** Summary of PAT-based modeling method applied in the drying process.

Equipment Type	Monitored IQAs in Process	PAT Tool (Model)	Related CQAs of Finished Product	Ref
Lyophilizer (Freeze-dryer)	- Particle state	Fourier transform-NIR spectrometer (ABB Bomem, Inc., Quebec, QC, Canada)	- Physical attributes - Degradation uniformity	[6]
	- Surface moisture content	NIR spectrometer system (Antaris, Thermo Electron, Madison, WI, USA)	- Moisture content - stability	[249]
	- Solid state - Release of hydrate	Raman spectrometer (RamanRxn1 spectrometer, Kaiser Optical Systems, Ann Arbor, MI, USA) NIR spectrometer (Thermo Fisher Scientific, Zellik, Belgium, Nicolet Antaris II near-IR analyzer)	- Physical attributes - Dissolution	[250]
	- Solid physicochemical changes	NIR spectrometer (Thermo Fisher Scientific, Nicolet Antaris II near-IR analyzer)	- Physical attributes - Stability	[251]
	- Moisture content - Solid state	Push-broom hyperspectral imaging system (VLNIR, Specim, Finland) Raman spectrometer (Rxn1 spectrometer, Kaiser Optical Systems, Ann Arbor, MI, USA)	- Physical attributes - Moisture content	[245]
	- Moisture content	NIR spectroscopy using an Fourier Transform spectrometer (Thermo Fisher Scientific, Madison, WI, USA)	- Moisture content	[158]
	- Residual moisture	RamanRxn1 spectrometer (Kaiser Optical Systems, Ann Arbor, MI, USA) NIR spectrometer (Thermo Fisher Scientific, Nicolet Antaris II near-IR analyzer) Cold plasma ionization device (Lyotrack 100, Adixen, France)	- Moisture content - Residual solvents	[252]
Fluidized-bed dryer	- Moisture content	Electrical capacitance tomography (ECT)	- Moisture content	[253]
	- Granule moisture	Microwave resonance technology, stray field resonator Hydorpharm (AMS Advanced Microwave Systems GmbH, Elmshorn, Germany)	- Moisture content	[254]
	- Drying endpoint - Residual moisture	Fourier-transform NIR spectrometer (Thermo Fisher Scientific, Zellik, Belgium, Nicolet Antaris II near-IR analyzer)	- Moisture content - Stability	[255]
